# Accumulation of uric acid in the epidermis forms the white integument of *Samia ricini* larvae

**DOI:** 10.1371/journal.pone.0205758

**Published:** 2018-10-15

**Authors:** Jung Lee, Takashi Kiuchi, Munetaka Kawamoto, Toru Shimada, Susumu Katsuma

**Affiliations:** Department of Agricultural and Environmental Biology, Graduate School of Agricultural and Life Sciences, The University of Tokyo, Yayoi, Bunkyo-ku, Tokyo, Japan; USDA Agricultural Research Service, UNITED STATES

## Abstract

The white color in the larval integument of the silkworm *Bombyx mori* is considered the result of uric acid accumulation in its epidermal cells. Larvae of the eri silkworm *Samia ricini* (Lepidoptera; Saturniidae) also have a white and opaque integument, but little is known about its coloration mechanism. In this study, we first performed a feeding assay of *S*. *ricini* larvae using allopurinol, an inhibitor of xanthine oxidase, which catalyzes the degradation of xanthine to uric acid. This treatment induced a clear translucent integument phenotype, indicating that the larval color of *S*. *ricini* is also determined by uric acid accumulation. Next, to investigate the genetic basis that controls uric acid accumulation in *S*. *ricini* larvae, we isolated and characterized the *S*. *ricini* homolog of mammalian *biogenesis of lysosome-related organelles complex 1*, *subunit 2* (*BLOS2*), which is known to play a crucial role in urate granule biosynthesis. We created a transcription activator-like effector nuclease (TALEN)-mediated gene knockout of *S*. *ricini BLOS2* (*SrBLOS2*) and succeeded in establishing *SrBLOS2* knockout strains (*SrBLOS2*^KO^). *SrBLOS2*^KO^ mutants exhibited a translucent larval integument phenotype and lacked uric acid in the epidermis, as also observed in allopurinol-fed larvae. In addition, electron microscopy revealed that urate granules were rarely observed in the epidermis of *SrBLOS2*^KO^ larvae, whereas abundant granules were found in the epidermis of wild-type larvae. These results clearly demonstrated that larval *S*. *ricini* accumulates uric acid as urate granules in the epidermis and that the genetic basis that controls uric acid accumulation is evolutionarily conserved in *S*. *ricini* and *B*. *mori*.

## Introduction

Uric acid is the final product of purine metabolism [1]. Most insects have been considered to merely emit uric acid to discard excessive nitrogen. However, recent studies revealed that insects reuse and utilize uric acid in diverse manners [2–3]. The larvae of *Bombyx mori* (Lepidoptera: Bombycidae) represent one example. *B*. *mori* larvae accumulate uric acid as urate granules in their epidermal cells [4–5]. As urate granules diffuse and reflect external light, the larval integument of *B*. *mori* appears white and opaque. Therefore, *B*. *mori* mutant strains with defects in uric acid synthesis, transport, or accumulation exhibit a translucent integument phenotype [6–8]. Although the biological significance of uric acid accumulation is unclear, it is widely accepted that urate granules in epidermal cells protect biomolecules against photooxidative stress. Uric acid is a physiological antioxidant [9], and uric acid-deficient *B*. *mori* larvae are hyper-susceptible to ultraviolet (UV) light and UV-induced reactive oxygen species [10–11]. Considering that the larvae of lepidopteran insects such as *Papilio polyxenes* and *Spodoptera litura*, the integuments of which are not entirely white, also accumulate uric acid [12–13], this theory appears highly plausible because wild insects are continuously exposed to photooxidative stress caused by natural UV light.

**Fig 1:**
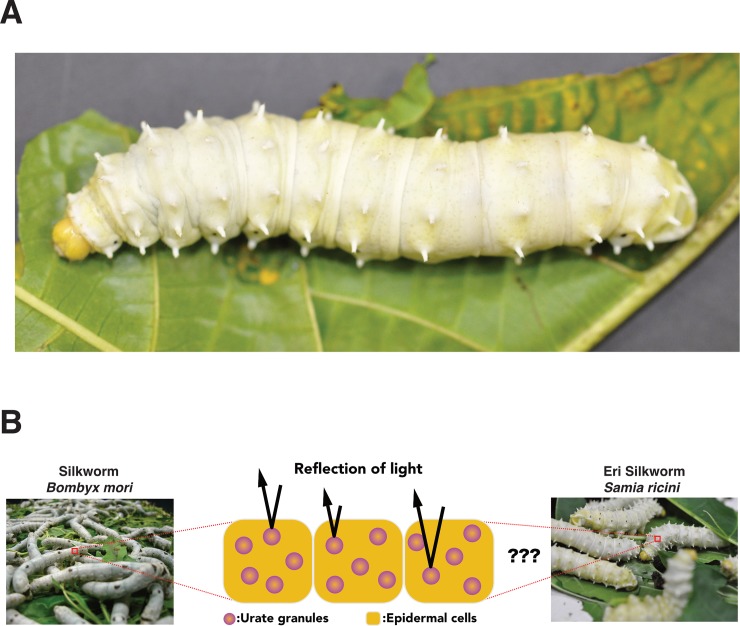
Graphical view of *Samia ricini* larva. (A) Fifth-instar larva of *S*. *ricini*. Its integument is white and opaque. (B) Coloration mechanism of the *Bombyx mori* larval integument (left) and a hypothetical model of *S*. *ricini* integument coloration (right).

Lepidopteran insects commonly accumulate uric acid in the epidermis, but it is still unclear that accumulated uric acid contributes the coloration of the larval integument in the lepidopteran species other than *B*. *mori*. *Samia ricini* (Lepidoptera; Saturniidae) is a gigantic silkworm with a white and opaque larval integument, similarly as *B*. *mori* (Fig 1A). If uric acid accumulation is responsible for the whiteness of the larval integument (Fig 1B), the deficiency of uric acid would cause a drastic change of its appearance. In this study, to reveal the coloration mechanism of the larval integument of *S*. *ricini*, we utilized both physiological and molecular biological approaches. First, we inhibited uric acid synthesis in *S*. *ricini* larvae via feeding with allopurinol, an inhibitor of xanthine oxidase, which catalyzes the degradation of xanthine to uric acid. Second, we performed transcription activator-like effector nuclease (TALEN)-mediated knockout (KO) of the homolog of a gene involved in uric acid accumulation in *B*. *mori*.

We focused on the *S*. *ricini* homolog of mammalian *biogenesis of lysosome-related organelles complex 1*, *subunit 2* (*BLOS2*) because *S*. *ricini BLOS2* (*SrBLOS2*) is likely a Z-linked gene due to the highly conserved synteny of the Z chromosome among lepidopteran insects [13–14]. By targeting *SrBLOS2*, we observed a mutant phenotype in generation 1 (G_1_) because G_1_ females can be hemizygous at the *SrBLOS2* locus.

In this study, we revealed that *S*. *ricini* larvae accumulate uric acid as urate granules in the epidermis and that a certain part of the genetic basis that controls uric acid accumulation is evolutionally conserved between *S*. *ricini* and *B*. *mori*. To the best of our knowledge, this is the first report of successful genome editing in *S*. *ricini*.

## Materials and methods

### Insects

*S*. *ricini* larvae were provided from National BioResource Project (NBRP; http://shigen.nig.ac.jp/wildmoth/). *S*. *ricini* larvae were reared on artificial diet (Insecta LFS, Nosan, Kanagawa, Japan) under a long-day condition (16 h light/8 h dark) at 25°C. TALEN-mediated gene KO larvae and their progenies (G_0_ and G_1_ individuals) were reared on *Ricinus communis* leaves under the same condition. Blue strain was one of the chromosome segment substitution lines, derived from backcrossing of F_1_ individuals between *S*. *ricini* and *Samia cynthia pryeri*, which is phylogenetically close species to *S*. *ricini* and also preserved in NBRP. Blue strain larvae were reared on *R*. *communis* under a short-day condition (12 h light/12 h dark) at 25°C.

### Inhibition of uric acid synthesis in *S*. *ricini* larvae

**Fig 2:**
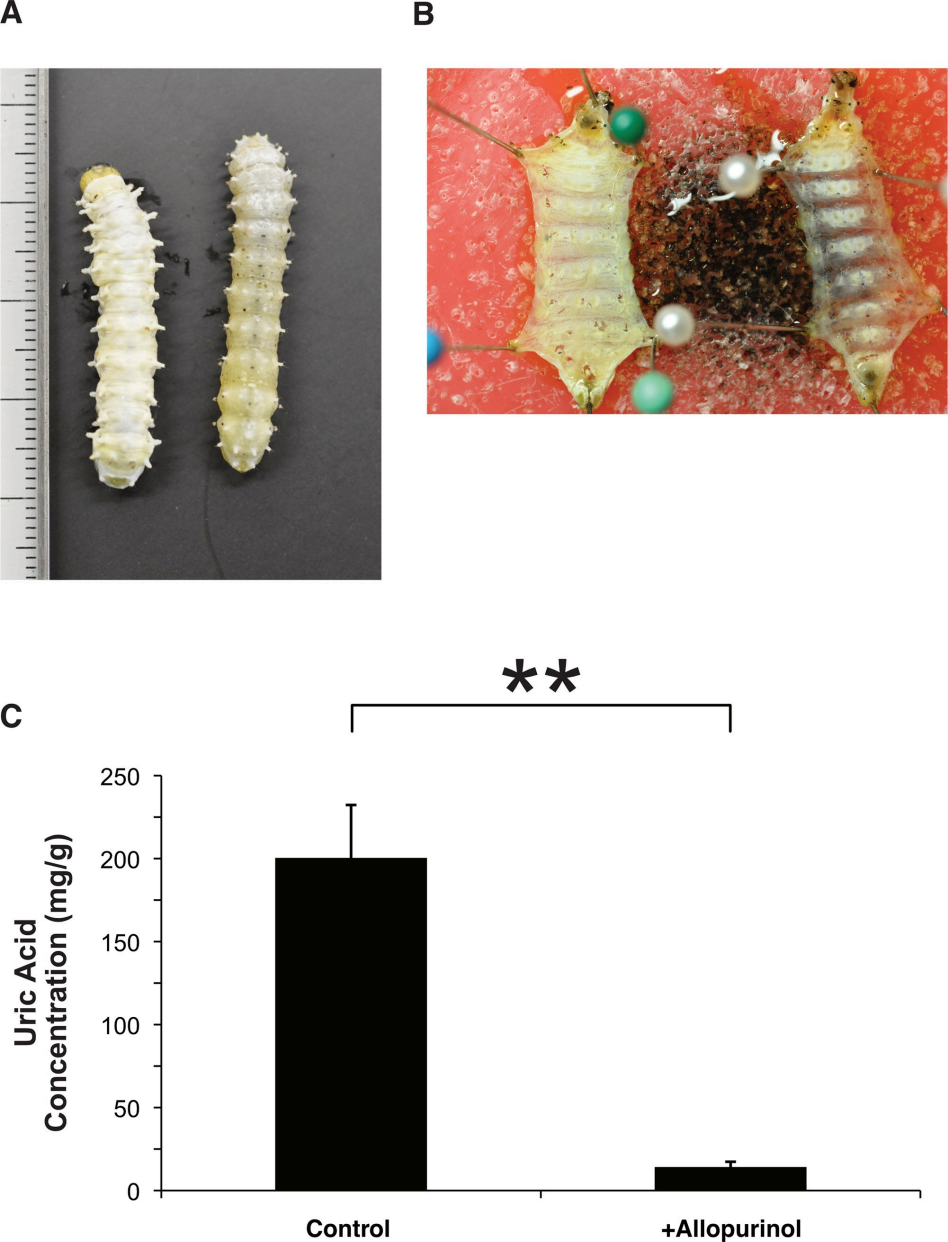
Phenotype of *Samia ricini* larvae treated with allopurinol. (A) Dorsal view of *S*. *ricini* larvae treated with (right) or without (left) allopurinol. (B) Ventral view of *S*. *ricini* larvae treated with (right) or without (left) allopurinol. To make observation easier, the ventral integument was dissected, and internal organs were removed. (C) Comparison of uric acid concentrations in the integument of *S*. *ricini* larvae treated with or without allopurinol. Data are shown as the means + standard error. n = 6. **p < 0.01 by Student *t*-test.

We used the method reported by Matsuo and Ishikawa (1999) [10]. Four hundred milligrams of allopurinol (Wako, Osaka, Japan) were added to 300 g of Insecta LFS and fed to *S*. *ricini* throughout the larval stage. The growth speed of individuals fed Insecta LFS is not uniform, making it impossible to collect individuals at the same developmental stage. In the experiments of Fig 2, we used individuals of the same age (20 days old).

### Quantification of uric acid content in the integuments of *S*. *ricini* larvae

The integuments of 20-day-old larvae or fifth-instar day 4 larvae were dissected and stored at −30°C until use. After homogenization, 0.002 g of the integuments were boiled in 1000 μL of distilled water for 10 min. The uric acid content was measured using a QuantiChrom Uric Acid Assay kit (BioAssay Systems, CA, USA) according to the manufacturer’s protocols. We measured the optical density of each sample at 595 nm using an iMARK Microplate Reader (BIO-RAD, CA, USA) and calculated the uric acid concentration of each sample. For measurements in *SrBLOS2*^KO^ mutants, strain #22 individuals were used.

### Determination of the partial nucleotide sequences of *SrBLOS2* and *SrRp49*

The partial nucleotide sequences of *SrBLOS2* and *SrRp49* cDNA were obtained through a tBLASTn search using *S*. *ricini* transcriptome data for the larval midgut (SilkBase, http://silkbase.ab.a.u-tokyo.ac.jp) with the amino acid sequences of *B*. *mori* BLOS2 (BmBLOS2, GenBank BAI63077.1) and BmRP49 (GenBank NP_001091752.1) as queries, respectively. In addition, to grasp the genomic structure of *SrBLOS2*, we designed three sets of primers to amplify its introns (S1 Table) according to the genomic structure of *BmBLOS2*. Genomic PCR was performed using KOD FX Neo (ToYoBo, Osaka, Japan). The PCR program for *SrBLOS2* was as follows: 98°C for 2 min; 40 cycles of 10 s at 98°C, 30 s at 57°C, and 3 min at 68°C; and 68°C for 2 min. Amplified fragments were cloned into pGEM-T Easy vectors (Promega, Madison, WI, USA) and sequenced on a 3130xl Genetic Analyzer (Applied Biosystems, Carlsbad, CA, USA). Sequence analyses were conducted using GENETYX-MAC version 16.0.1 (GENETYX Co., Tokyo, Japan) and ATSQ version 5.1.3 (GENETYX Co.).

### Phylogenetic analysis

The phylogenetic tree of insect BLOS2 homologs was constructed using MEGA7.0 [15]. The evolutionary history was inferred using the maximum likelihood method based on the JTT matrix-based model [16]. The tree with the highest log likelihood (−1293.7573) was shown. The percentage of trees in which the associated taxa clustered together was calculated via bootstrapping with 1000 replicates. To generate a phylogenetic tree, we used 78 amino acid sequences, including 12 sequences from lepidopteran insects, 3 sequences from hemipteran insects, 27 sequences from dipteran insects, 7 sequences from coleopteran insects, 28 sequences from hymenopteran insects, and 1 sequence from *Homo sapiens*. The GenBank accession numbers of these amino acid sequences are listed in S2 Table.

### Expression analysis of *SrBLOS2* mRNA in *S*. *ricini* tissues

Total RNA was extracted from *S*. *ricini* embryos or tissues using TRIzol reagent (Invitrogen, Carlsbad, CA, USA) according to the manufacturer’s protocol and subjected to reverse transcription using SuperScript III reverse transcriptase (Thermo Fisher Scientific, MA, USA) with oligo-dT primers (TaKaRa Bio, Shiga, Japan). RT-PCR was performed using KOD FX Neo (ToYoBo) or Ex-Taq (TaKaRa). The primers used are listed in S1 Table. The PCR program for *SrBLOS2* was as follows: 98°C for 2 min; 40 cycles of 10 s at 98°C, 30 s at 60°C, and 15 s at 68°C; and 68°C for 2 min. The PCR program for *SrRp49* was as follows: 94°C for 2 min; 25 cycles of 20 s at 94°C, 30 s at 60°C, and 30 s at 72°C; and 72°C for 10 min. The amplified fragments of *SrBLOS2* and *SrRp49* were cloned into pGEM-T Easy vectors and sequenced on a 3130xl Genetic Analyzer. Sequence analyses were conducted using GENETYX-MAC version 16.0.1 and ATSQ version 5.1.3.

### Construction of a TALEN targeting *SrBLOS2*

A pair of TALENs was designed to target the coding sequence of *SrBLOS2* using TALEN Targeter (https://tale-nt.cac.cornell.edu/node/add/talen). We located the target site, the sequence of which was 5′-CCAGCTTTGAAGTACTGGAtccacatgaccctgtTATAAGTAGGTTAGCAACTC-3′, on exon 2. The nucleotides in capital letters indicate the RVD binding sites of the left and right TALENs. The TALE modules were assembled using Golden Gate TALEN and TAL Effector kit 2.0 (Addgene, Cambridge, MA, USA) in accordance with the highly efficient construction methods developed by Cermak et al. (2011) with some modifications by Takasu et al. (2014) [17,18]. pBlue-TAL (GenBank accession no. KF724948), which was developed for genome editing in *B*. *mori* [18], was used as a destination plasmid. The TALEN mRNA was synthesized in vitro using a MEGAscript T7 ultra Kit (Ambion, MA, USA) according to the manufacturer’s protocols. Purified mRNA was dissolved in annealing buffer (100 mM potassium acetate, 2 mM magnesium acetate, 30 mM 4-(2-hydroxyethyl)-1-piperazineethanesulfonic acid-KOH pH 7.4), adjusted to three different concentrations (200, 400, and 600 ng/μL), and stored at −80°C until use. Immediately before embryonic injection, left and right TALEN mRNAs were mixed.

### Embryo preparation and embryonic microinjection

To allow *S*. *ricini* moths to copulate, pairs of male and female moths were placed in small paper bags. After 18–24 h of copulation, the pairs were decoupled, and males were removed. Female moths started laying eggs on the wall of the paper bag immediately when the dark period began.

Eggs detached from the paper bag were individually affixed onto a microscope slide using instant glue. Injection was performed using a microinjector (IM 300 Microinjector, Narishige, Tokyo, Japan). Approximately 1–5 nL of each TALEN mRNA solution were injected into each embryo between 4–8 h after oviposition. All procedures were performed at 25°C. The injected embryos were incubated at 25°C in a humidified Petri dish, which promoted their hatching in 10 days.

### Crossing of G_0_ individuals

Adult G_0_ moths were crossed with each other. After virgin individuals of the opposite sex were used up, G_0_ moths were crossed with wild-type moths. After 36–48 h of copulation, the pairs were decoupled, and males were removed. All of the parents of the three strains described in the Results section were G_0_ individuals.

### Detection and DNA sequencing of the mutations in G_1_ individuals

Ten newly hatched larvae from each brood were collected in one tube, and genomic DNA was extracted using the HotSHOT method [19]. This step was repeated on the other set of 10 newly hatched larvae. Genomic PCR was performed using KOD FX Neo (ToYoBo) with specific primers (S1 Table). PCR products were diluted 2-fold with 1× TE buffer, denatured, and hybridized as follows: 95°C for 10 min; ramping down at −2°C/s to 85°C and at −0.1°C/s to 25°C; and finally holding at 4°C. Microchip electrophoresis of the re-hybridized PCR products was performed using MultiNA (Shimadzu, Kyoto, Japan). The PCR products of the *SrBLOS2* locus were also used for DNA sequencing after being cloned into pGEM-T Easy vectors. For each brood, 20 bacterial colonies were selected and analyzed by DNA sequencing. Regarding strain #28, genomic DNA was extracted from three G_2_ male larvae using DNeasy Blood & Tissue kit (QIAGEN, Hilden, Germany) and used for DNA sequencing of their *SrBLOS2* loci.

### Transmission electron microscopy (TEM)

The integuments of fifth-instar day 4 larvae (*SrBLOS2*^KO^ and wild-type) were dissected, fixed for 24 h at 4°C in 4% paraformaldehyde in phosphate buffer solution, and washed twice with phosphate buffer solution for 10 min on ice. Secondary fixation was performed for 3 h at 4°C in 2% osmium (VIII) oxide in phosphate buffer solution, and specimens were washed twice with phosphate buffer solution for 10 min on ice. After fixation, the tissues were dehydrated in a graded ethanol series. In the final step of dehydration, the tissues, soaked in absolute ethanol, were placed at 4°C for overnight. Embedding was performed using a Spurr Low Viscosity Embedding Kit (Polysciences, Warrington, PA, USA) according to the manufacturer’s protocols. Polymerization was performed at 70°C for 24 h. Eighty-nanometer-thick sections were excised and stained with 4% uranyl acetate and lead citrate. The sections were examined at 80 kV under a transmission electron microscope (JEM-1400 plus, Nihondenshi, Tokyo, Japan).

### Nucleotide sequence deposition

The partial cDNA sequences of *SrBLOS2* and *SrRp49* and DNA sequences of three introns of *SrBLOS2* are available under the accession numbers LC378373, LC378374, and LC378375– LC 378377, respectively.

## Results

### Allopurinol treatment induced a translucent integument phenotype in *S*. *ricini* larvae

Compared with the control group, the integument of the allopurinol-treated group exhibited a slightly translucent phenotype (Fig 2A and 2B). The uric acid concentration was drastically reduced in the integument of larvae in the allopurinol-treated group (Fig 2C). These results indicate that the white color of the *S*. *ricini* larval integument is formed by the accumulation of uric acid in the epidermis.

### Identification and characterization of *SrBLOS2*

Through a BLAST search against *S*. *ricini* midgut transcriptome data, we identified a contig that includes a putative coding sequence (CDS) of *S*. *ricini BLOS2* (*SrBLOS2*). The *SrBLOS2* CDS is 438 base pairs in length, and an InterProScan search of the deduced amino acid sequence revealed that SrBLOS2 belongs to the BLOS2 family (IPR019269).

**Fig 3:**
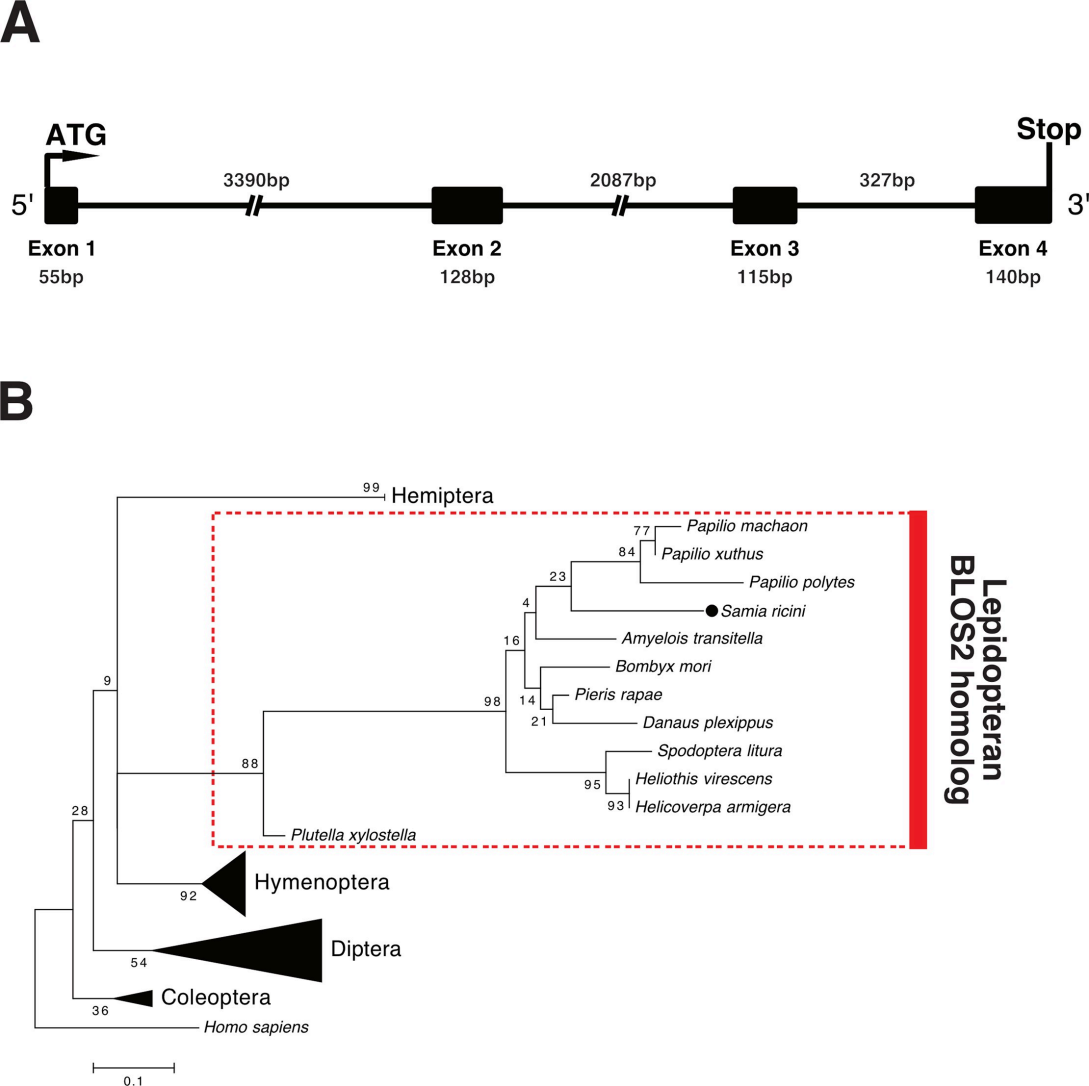
Genomic structure and phylogenetic analysis of *Samia ricini biogenesis of lysosome-related organelles complex 1*, *subunit 2* (*SrBLOS2*). (A) Genomic structure of *SrBLOS2*. Numbers show the sizes of exons and introns. (B) Phylogenetic tree of insect BLOS2 homologs. The tree was constructed using MEGA7.0 (Kumar et al., 2015). Human BLOS2 sequence was included as an outgroup.

We designed three sets of primers to amplify the intronic regions of *SrBLOS2* using the genomic structure of *BmBLOS2* as a reference. Genomic PCR revealed that the *SrBLOS2* CDS consists of four exons (Fig 3A). In addition, phylogenetic analysis revealed that lepidopteran BLOS2 homologs including *SrBLOS2* are clustered into a single clade (Fig 3B), indicating that *SrBLOS2* is an ortholog of lepidopteran *BLOS2*.

### Expression profile of *SrBLOS2* in larval tissues

**Fig 4:**
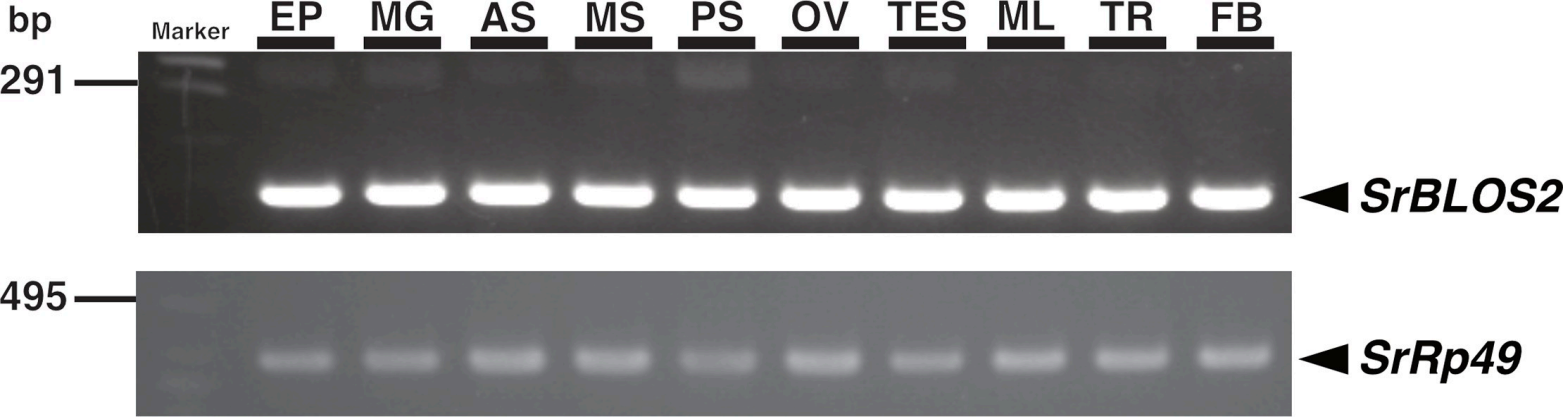
RT-PCR analysis of *Samia ricini biogenesis of lysosome-related organelles complex 1*, *subunit 2* (*SrBLOS2*) in *S*. *ricini* larval tissues. Total RNA of fifth-instar day 4 larvae of the wild-type individuals was used for RT-PCR. *SrRp49* was used as an internal control. EP, epidermis; MG, midgut; AS, anterior silk gland; MS, middle silk gland; PS, posterior silk gland; OV, ovary; TES, testis; ML, Malpighian tubule; TR, trachea; FB, fat body.

To investigate in which tissue *SrBLOS2* functions, *SrBLOS2* expression in 10 larval tissues was examined by RT-PCR. In fifth-instar day 4 larvae, *SrBLOS2* expression was detected in all examined tissues (Fig 4). This ubiquitous expression pattern was similar to that of *BmBLOS2* [7].

### Microinjection of the TALEN mRNA for *SrBLOS2*

**Fig 5:**
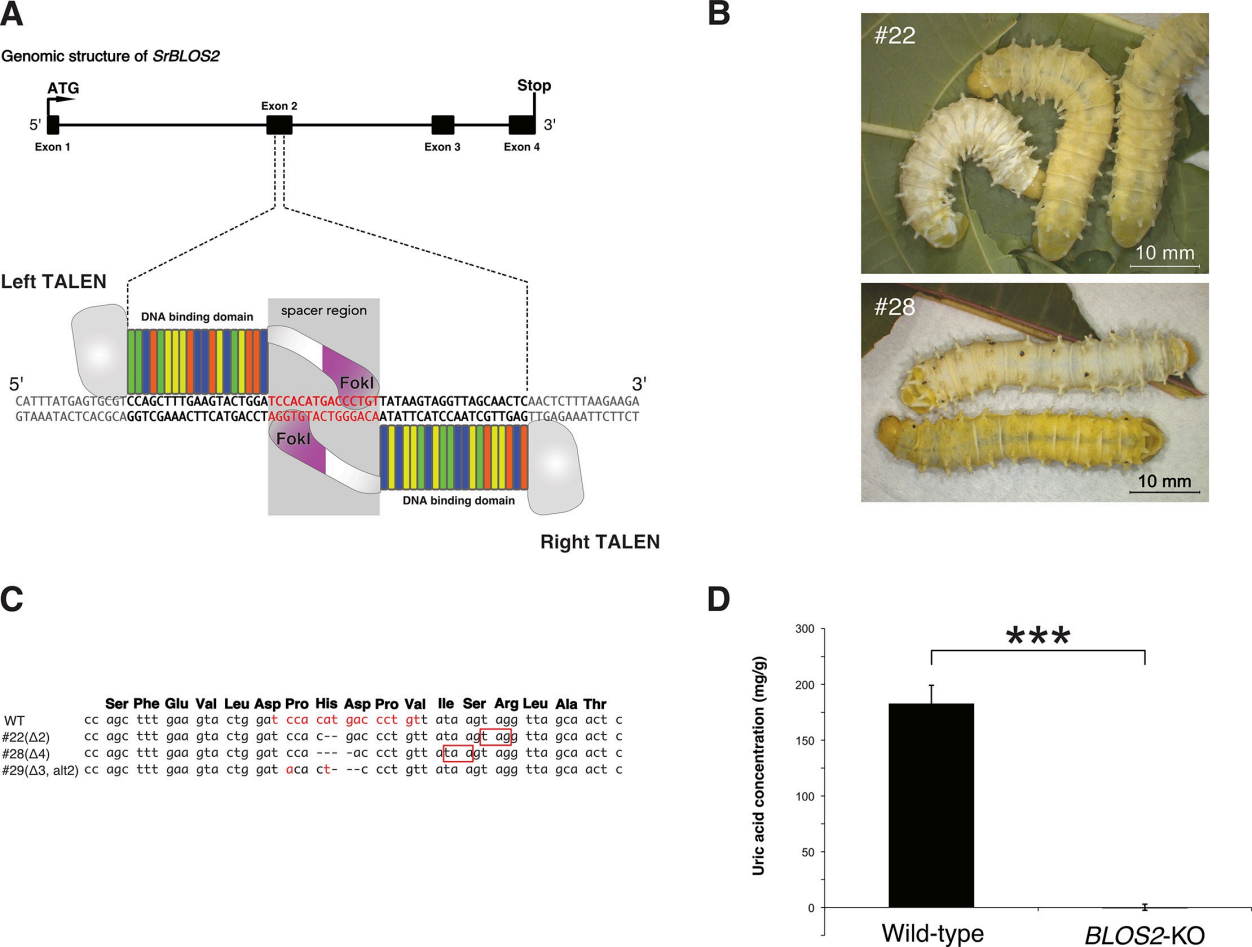
Characterization of transcription activator-like effector nuclease (TALEN)-generated *Samia ricini biogenesis of lysosome-related organelles complex 1*, *subunit 2* knockout (*SrBLOS2*^KO^) mutants. (A) Schematic presentation of the spacer and binding sequences of TALEN targeting *SrBLOS2*. (B) Larval phenotype of *SrBLOS2*^KO^ individuals. The upper panel shows larvae from strain #22. A wild-type larva is shown on the left, and two translucent individuals are presented on the right. The lower panel shows larvae from strain #28. A wild-type larva is presented on the top, and one translucent individual is shown on the bottom. (C) Mutations introduced in three *SrBLOS2*^KO^ strains. The spacer sequence is shown in red. Premature stop codons generated by frame shift are highlighted with red squares. The deduced amino acid sequence of wild-type *SrBLOS2* is shown on the top. (D) Comparison of uric acid concentrations in the integument of *S*. *ricini* wild-type and strain #22 larvae. Data are shown as the mean + standard error. n = 3. ***p < 0.001 by Student’s t-test.

We designed the targeting site of TALEN on exon 2 of the *SrBLOS2* CDS (Fig 5A). The left and right TALEN mRNA mixture at concentrations of 400 (200 + 200), 800 (400 + 400), and 1200 (600 + 600) ng/μL was injected to three batches of eggs (144, 312, and 164 eggs, respectively). The injected eggs displayed hatching ratios of 20.8 (30/144), 40.1 (125/312), and 22.6% (37/164), respectively (Table 1). Although we expected to observe somatic mosaics in the integument of G_0_, we failed to identify individuals exhibiting the mottled and translucent phenotype in their integument.

**Table 1:** Efficiency of transcription activator-like effector nuclease-mediated knockout of *Samia ricini biogenesis of lysosome-related organelles complex 1*, *subunit 2*.

TALEN mRNA concentration (Left TALEN + Right TALEN)	No. of injected embryos	No. of hatched	No. of 5th instar larvae	No. of G1 broods carrying mutant alleles	Germline transmission rate (%)
200 ng/μL + 200 ng/μL	144	30	20	0	0
400 ng/μL + 400 ng/μL	312	125	79	3	3.80
600 ng/μL + 600 ng/μL	164	37	29	0	0

We performed microinjection of left and right TALEN mRNA at three different concentrations (200 ng/μL, 400 ng/μL, 600 ng/μL). The columns are: No. of injected embryos–number of embryos that are used for microinjection; No. of hatched–number of hatched embryos that survived after microinjection; No. of 5^th^ instar larvae–number of larvae that normally grew up to 5^th^ instar; No. of G1 broods carrying mutant alleles–number of G_0_ individuals whose progenies have mutated *SrBLOS2* alleles; Germline transmission rate–values calculated from 4^th^ and 5^th^ columns.

### Detection of mutant alleles in G_1_ broods

By crossing the sibling G_0_ individuals with each other, we obtained 128 G_1_ broods (Table 1). Among them, 20, 79, and 29 were obtained from the egg batches injected with 400, 800, and 1200 ng/μL TALEN mRNA, respectively. Through microchip electrophoresis, we found that two G_1_ broods (strains #22 and #29) had mutations in the *SrBLOS2* locus (S1 Fig). Of these, only strain #22 included individuals exhibiting the translucent integument phenotype (Fig 5B). In addition, strain #28, in which the mutation was not detected by microchip electrophoresis, contained individuals with the translucent integument phenotype (Fig 5B).

Based on the microchip electrophoresis results, the mutations of strains #22 and #29 were transmitted from either of the parents (S1A and S1B Fig). If different mutations were inherited from both parents, there should have been more heteroduplex bands. Concerning strain #28, translucent G_1_ individuals were all female, and G_2_ progenies obtained by sibling crossing did not exhibit a translucent phenotype, indicating that the G_0_ founder was male.

DNA sequencing of the *SrBLOS2* loci of the three strains revealed that each strain had a single different mutant allele. A two-nucleotide deletion, a four-nucleotide deletion, and a three-nucleotide deletion and two-nucleotide substitution were detected in strains #22, #28, and #29, respectively. The former two alleles generated a premature stop codon, whereas the latter generated a single amino acid deletion and two amino acid substitutions (Fig 5C).

### Germline transmission rates (GTRs) of TALEN-mediated *SrBLOS2* mutations

We calculated the GTR as the number of G_1_ broods with mutant alleles divided by the number of total G_0_ adult moths. The GTR of TALEN-mediated *SrBLOS2* mutation was 3.80% (Table 1).

### Accumulation of uric acid as urate granules in the epidermal cells of *S*. *ricini* larvae

To confirm whether the translucent integument phenotype of TALEN-mediated *SrBLOS2*^KO^ mutants was due to the lack of urate granules, we measured the uric acid concentration in the epidermis of *SrBLOS2*^KO^ mutants. Compared with wild-type individuals, the mutants almost completely lacked uric acid accumulation in their epidermis (Fig 5D).

**Fig 6:**
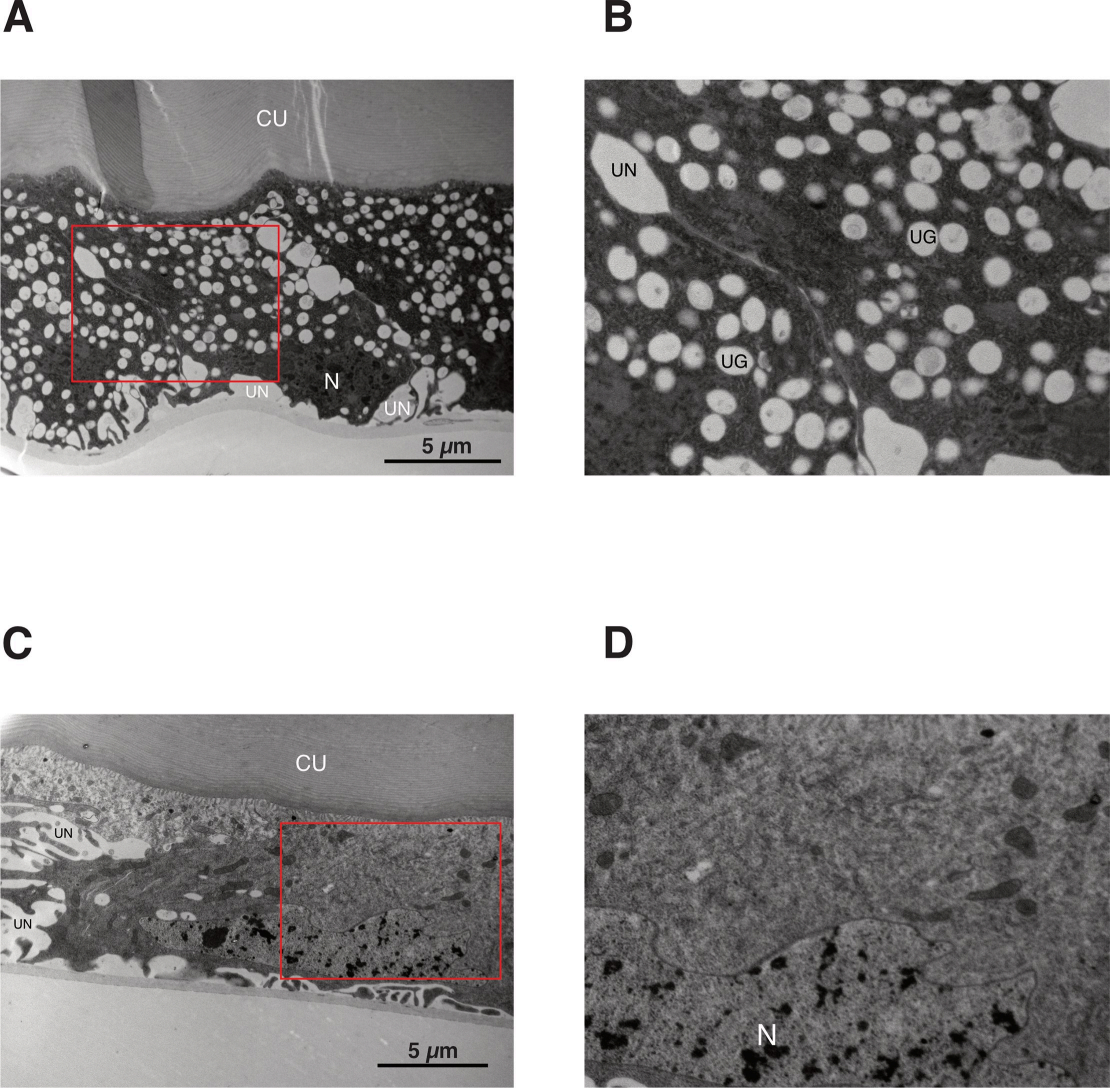
Transmission electron microscopy of the epidermis of wild-type and *Samia ricini biogenesis of lysosome-related organelles complex 1*, *subunit 2* knockout (*SrBLOS2*^KO^) individuals. Low- (A) and high-magnification (B) micrographs of the epidermis of a fifth-instar day 4 wild-type larva. Low- (C) and high-magnification (D) micrographs of the epidermis of a fifth-instar day 4 *SrBLOS2*^KO^ larva. Red squares in A and C indicate the regions enlarged in B and D, respectively. N, nucleus; CU, cuticle; UG, urate granules; UN, unknown vacuole-like organelles.

Furthermore, TEM revealed that the epidermal cells of wild-type individuals had numerous oval-shaped urate granules (Fig 6A and 6B), whereas *SrBLOS2*^KO^ mutants had an extremely reduced number of such granules (Fig 6C and 6D), indicating that *SrBLOS2* is indispensable for the formation of urate granules in the epidermal cells of *S*. *ricini* larvae.

## Discussion

### Accumulation of uric acid in the integument of *S*. *ricini* larvae

Because the integument of the allopurinol-treated individuals and *SrBLOS2*^KO^-mutants were colorless and translucent (Figs 2 and 5B), we concluded that uric acid is the major substance that forms the uniformly white integument of *S*. *ricini* larvae. Conversely, the majority of Saturniidae species exhibit various patterns of larval integument coloration. As represented by *Antheraea yamamai* (Japanese oak silkmoth) or *Antheraea pernyi* (Chinese oak silkmoth), the basal color of other saturniid larval integuments is green, which is generated by a mixture of bilins and carotenoids [20]. In these species, uric acid does not likely contribute to the green integument color. Some strains of *S*. *ricini* have blue integuments (S2A Fig, “blue” strain). However, the blue strains contain a similar amount of uric acid in their epidermis (S2B Fig). Considering the result that *Spodoptera litura*, the larval integument of which is uniformly black regardless of the body part, also accumulates uric acid [13], it is concluded that uric acid accumulation is not always correlated with the integument color of lepidopteran larvae.

### *SrBLOS2* functions in urate granule formation

Some *B*. *mori* strains display a translucent integument phenotype. Among these mutant strains, *od* (*d**istinct* *o**ily*), the responsible gene of which has been elucidated as *BmBLOS2*, exhibits relatively high translucency [7, 21]. TEM revealed that *od* mutants accumulate reduced numbers of smaller urate granules compared with those observed in wild-type larvae [5].

BLOS2 is one of eight subunits of the BLOC-1 complex [22], which is required for normal biogenesis and trafficking of lysosome-related organelles [23]. The BLOC-1 complex is considered to function at an early stage of the melanosome, as BLOC-1 deficiency is associated with a bleached coat color in mice and red eye in humans [24]. Given these previous reports, it is extremely likely that BmBLOS2 functions in the formation of urate granules. In the present study, we observed that *SrBLOS2*^KO^ mutants also exhibited a translucent integument, deficient uric acid accumulation, and a reduced number of urate granules in the epidermis (Figs 5D and 6), indicating that the function of BLOS2 is conserved between *B*. *mori* and *S*. *ricini*. In addition, as reported in *BmBLOS2* and *S*. *litura BLOS2* [7, 13], *SrBLOS2* was found to be located on the Z chromosome. Compared with the autosomal genes, morphological phenotypes of KO mutants can be observed one generation earlier, demonstrating that the *BLOS*2 homologs represent ideal targets for applying genome-editing approaches to non-model lepidopteran species.

### Absence of *SrBLOS2*^KO^ G_0_ individuals with a somatic mosaic phenotype

We failed to observe somatic mosaics in the integument of *SrBLOS2*^KO^ G_0_ individuals. When *S*. *ricini* larvae enter the third instar, they begin to produce a crystalline white powder from spines on their backs [25]. This powder spreads from the spines and covers the entire larval body. Production of the white powder stops during the molting period, but other than that period, a huge amount of white powder is produced, making it impossible to distinguish *SrBLOS2*^KO^ mutants and wild-type individuals visually. For this reason, we did not find G_0_ mosaic larvae even though their mosaic phenotype appeared in the integument. Unlike the larval integument, the crystalline powder is not composed of uric acid, as its major components are straight chain-saturated alcohols, namely *n*-triacontanol (C_30_H_62_O, 92.0%) and *n*-octacosanol (C_28_H_58_O, 0.56%) [25]. Through TEM, vacuole-like large organelles other than urate granules were discovered (Fig 6B). Because similar organelles were not observed in the larval integuments of *B*. *mori* [5], these vacuole-like organelles were likely *S*. *ricini*-specific. These organelles appeared to emerge from the basement membrane of the epidermal cells (Fig 6B) and existed even in the epidermal cells of *SrBLOS2*^KO^ mutants (Fig 6D). The large size of these organelles might imply their role in transporting or synthesizing chemical compounds such as *n*-triacontanol or *n*-octacosanol.

### GTR of TALEN-mediated mutations in *S*. *ricini*

Although Takasu et al. [18] reported that the GTR of TALENs that induced somatic mosaics in G_0_ individuals reached 100% in *B*. *mori*, that of TALENs in *S*. *ricini* was extremely low (3.80%, Table 1). Previous studies reported that the GTR was highly dependent on target sequences regardless of the genome-editing tools. For example, Yang et al. [26] designed two TALEN pairs to knock out *odorant receptor co-receptor* in *O*. *furnacalis*. Although one TALEN pair was highly efficient and the GTR reached 62.9%, the other pair did not induce the generation of even somatic mosaics. Thus, the GTR of TALENs in *S*. *ricini* might be improved by changing the target sequence. We also suspect that the structure of the TALEN expression vector pBlue-TAL also contributed to the low GTR observed in *S*. *ricini*. pBlue-TAL is customized for mRNA translation in *B*. *mori*; i.e., codon usage, the Kozak sequence, and the UTR track were optimized on the basis of the genomic information of *B*. *mori* [18]. When the genome of *S*. *ricini* is sequenced in the future, we will be able to customize pBlue-TAL for *S*. *ricini* and improve the GTR of TALEN.

As their large bodies are suitable for experimentation, entomologists have often used *S*. *ricini* as a model for physiological and biochemical research [27–29], and knowledge and experimental techniques for this species have accumulated [30–31]. To increase the utility of *S*. *ricini* as a model organism, the whole-genome sequencing of *S*. *ricini* is strongly expected.

## Supporting information

S1 Table Primer list used in this study. (XLSX)

S2 Table GenBank accession number list of sequences used for phylogenetic analysis. (XLSX)

S1 Fig Representative images of microchip electrophoresis. (A) Detection of mutations introduced in generation 1 broods. Their parents are from eggs injected with 800 (400 + 400) ng/μL transcription activator-like effector nuclease mRNA. PCR failed in the second lot of strains #29 and #30.
(B) Higher-magnification images of microchip electrophoresis of strains #22 and #29, which include *Samia ricini biogenesis of lysosome-related organelles complex 1*, *subunit 2* (*SrBLOS2*^KO^) mutants. In addition to the major band (indicated by arrows), two bands (indicated by asterisks) were detected, indicating that the PCR products from *SrBLOS2* of strains #22 and #29 are heterozygous.
(TIFF)

S2 Fig The blue strain of *Samia ricini*. (A) Fifth-instar larvae of *S*. *ricini* immediately before the spinning stage. The color of its integument is slightly bluish.
(B) Comparison of uric acid concentrations in the integument of wild-type and blue strains of *S*. *ricini*. Data are shown as the mean + standard error. N.S., *p* > 0.05 by Student’s t-test.
(TIFF)
