# Supplementary figures and images for: Accumulation of uric acid in the epidermis forms the white integument of Samia ricini larvae
Source: PLoS One. 2018 Oct 15;13(10):e0205758. doi: 10.1371/journal.pone.0205758 (PMC6188861; doi:10.1371/journal.pone.0205758)

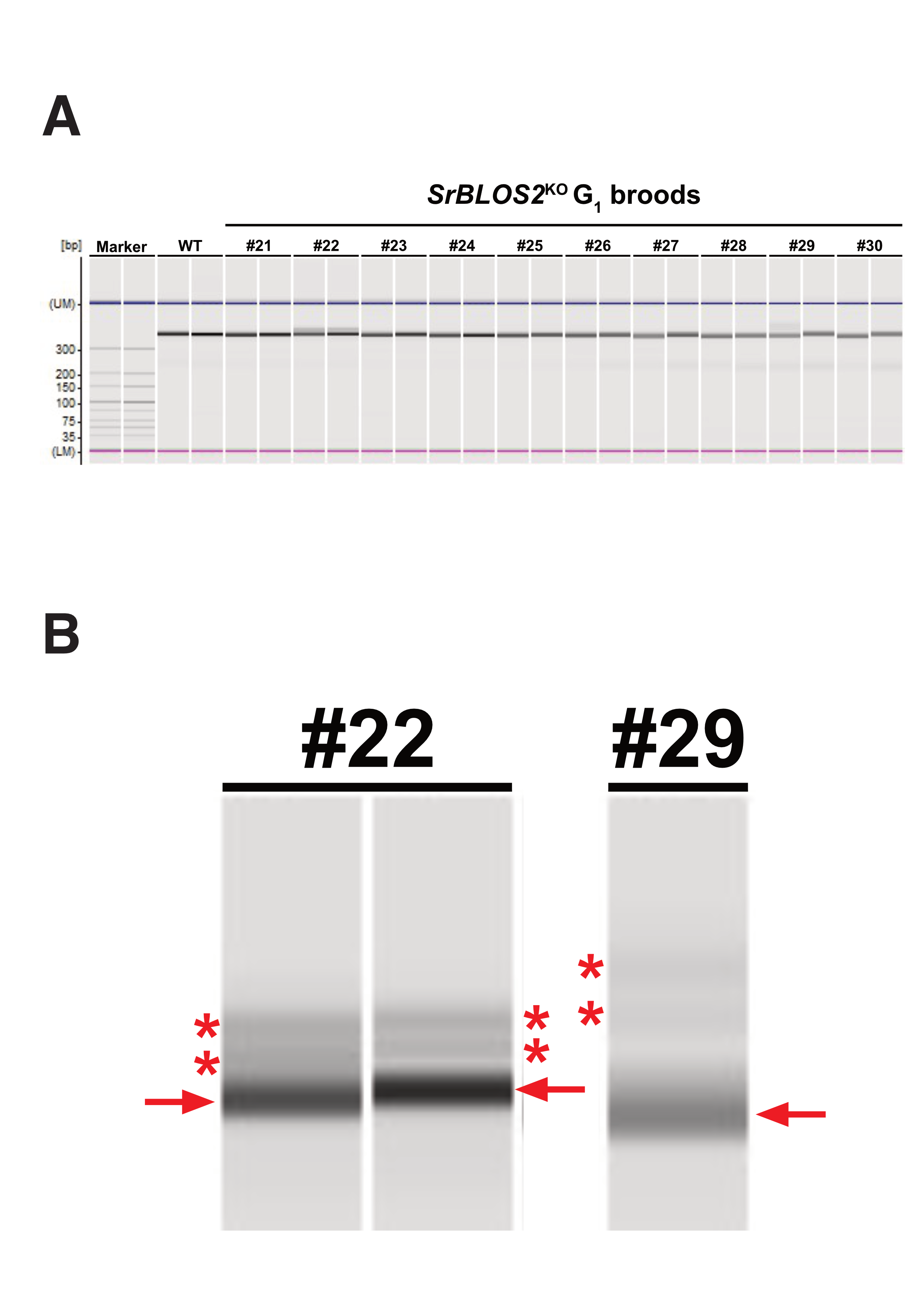

Supplement: S1 Fig — (A) Detection of mutations introduced in generation 1 broods. Their parents are from eggs injected with 800 (400 + 400) ng/μL transcription activator-like effector nuclease mRNA. PCR failed in the second lot of strains #29 and #30. (B) Higher-magnification images of microchip electrophoresis of strains #22 and #29, which include Samia ricini biogenesis of lysosome-related organelles complex 1, subunit 2 (SrBLOS2KO) mutants. In addition to the major band (indicated by arrows), two bands (indicated by asterisks) were detected, indicating that the PCR products from SrBLOS2 of strains #22 and #29 are heterozygous. (TIFF) [file pone.0205758.s003.tiff]

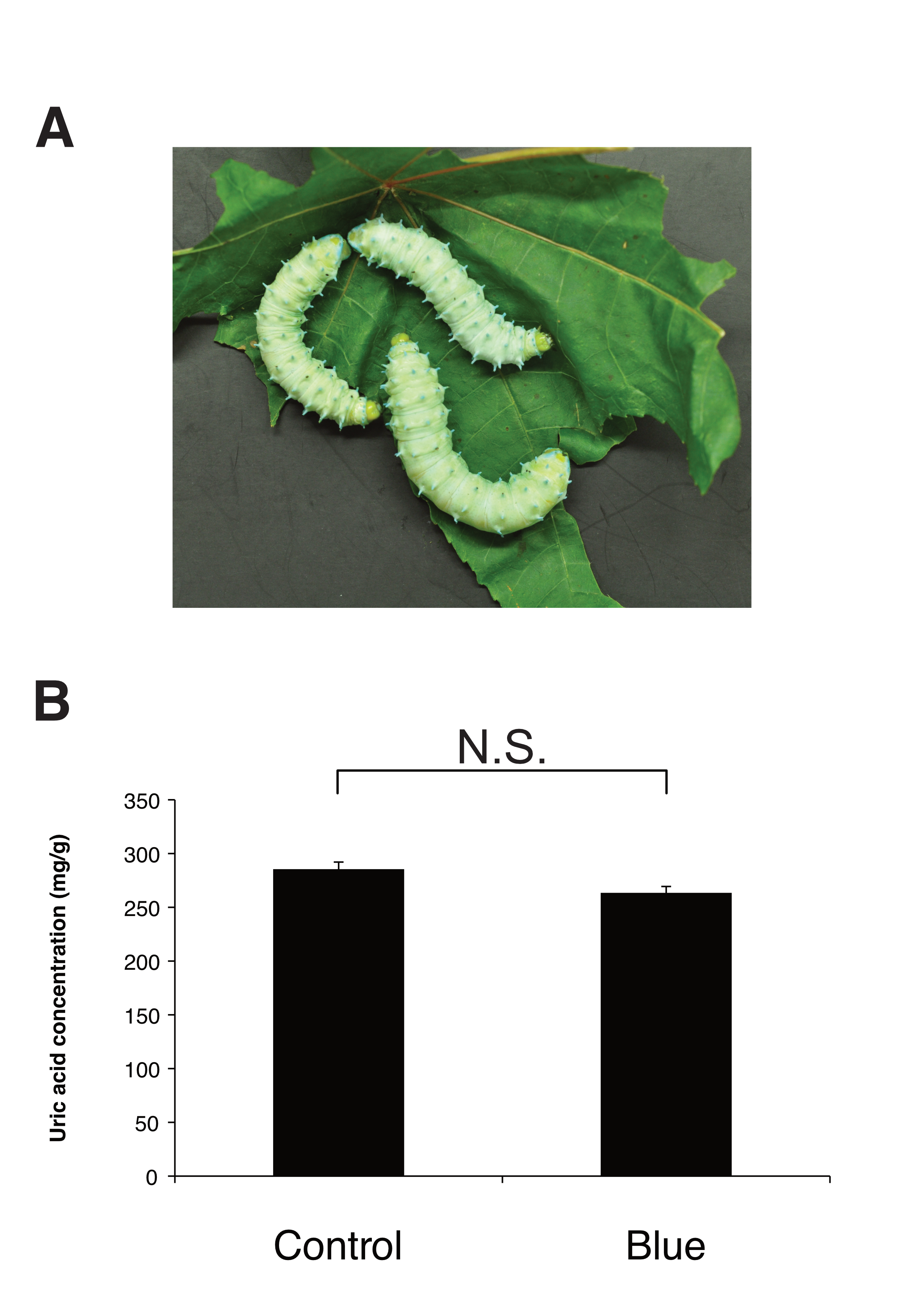

Supplement: S2 Fig — (A) Fifth-instar larvae of S. ricini immediately before the spinning stage. The color of its integument is slightly bluish. (B) Comparison of uric acid concentrations in the integument of wild-type and blue strains of S. ricini. Data are shown as the mean + standard error. N.S., p > 0.05 by Student’s t-test. (TIFF) [file pone.0205758.s004.tiff]
